# Network-Based Analysis of OMICs Data to Understand the HIV–Host Interaction

**DOI:** 10.3389/fmicb.2020.01314

**Published:** 2020-06-17

**Authors:** Sergey Ivanov, Alexey Lagunin, Dmitry Filimonov, Olga Tarasova

**Affiliations:** ^1^Department of Bioinformatics, Institute of Biomedical Chemistry, Moscow, Russia; ^2^Department of Bioinformatics, Pirogov Russian National Research Medical University, Moscow, Russia

**Keywords:** virus–host interaction, human immunodeficiency virus, protein–protein interactions, OMICs, transcriptomics, network analysis

## Abstract

The interaction of human immunodeficiency virus with human cells is responsible for all stages of the viral life cycle, from the infection of CD4+ cells to reverse transcription, integration, and the assembly of new viral particles. To date, a large amount of OMICs data as well as information from functional genomics screenings regarding the HIV–host interaction has been accumulated in the literature and in public databases. We processed databases containing HIV–host interactions and found 2910 HIV-1-human protein-protein interactions, mostly related to viral group M subtype B, 137 interactions between human and HIV-1 coding and non-coding RNAs, essential for viral lifecycle and cell defense mechanisms, 232 transcriptomics, 27 proteomics, and 34 epigenomics HIV-related experiments. Numerous studies regarding network-based analysis of corresponding OMICs data have been published in recent years. We overview various types of molecular networks, which can be created using OMICs data, including HIV–human protein–protein interaction networks, co-expression networks, gene regulatory and signaling networks, and approaches for the analysis of their topology and dynamics. The network-based analysis can be used to determine the critical pathways and key proteins involved in the HIV life cycle, cellular and immune responses to infection, viral escape from host defense mechanisms, and mechanisms mediating different susceptibility of humans to infection. The proteins and pathways identified in these studies represent a basis for developing new anti-HIV therapeutic strategies such as new drugs preventing infection of CD4+ cells and viral replication, effective vaccines, “shock and kill” and “block and lock” approaches to cure latent infection.

## Introduction

Human immunodeficiency virus (HIV) is one of the most significant pathogens to affect humankind. According to the World Health Organization, approximately 37.9 million people are currently living with HIV, and 770,000 people died from HIV-related disorders in 2018. Although the existing combination antiretroviral therapy (cART) provides control of the virus and prevents transmission, the HIV infection remains a global health problem (World Health Organization, 2020). Thus, there is an urgent need to understand anti-HIV mechanisms, to develop new strategies for its prophylactics and therapy.

The HIV infection process involves several stages from the binding of virions to receptors on the human CD4+ cell surface to the splicing and export of viral mRNAs from the nucleus to the cytoplasm, and the assembly of virions at the plasma membrane as well as the budding and maturation of the released virions (Kirchhoff, 2013; Chen et al., 2018). Like other viruses, HIV cannot complete any aspect of its life cycle without interacting with the host cellular machinery, primarily human proteins and various types of RNA. The human macromolecules, which are required for various stages of HIV life cycle, called host dependency factors (HDFs). Macromolecules, which are part of cell defense mechanisms and prevent viral infection, called host restriction factors (HRFs). During the HIV attack, several HRFs interfere with viral replication at different steps (Shukla and Chauhan, 2019). For instance, cytidine deaminase, APOBEC3G (apolipoprotein B mRNA editing enzyme, catalytic polypeptide-like 3G) induces lethal hypermutations (deamination of C to U) in the HIV genome, which are detrimental to viral replication. For more information on HDFs and HRFs see the reviews (Yamashita and Engelman, 2017; Chen et al., 2018; Engelman and Singh, 2018; Shukla and Chauhan, 2019).

Most of the existing anti-HIV drugs are inhibitors of three HIV enzymes, namely protease, reverse transcriptase and integrase; however, researchers are also studying HDFs as new potential targets (Arhel and Kirchhoff, 2010; Zhan et al., 2016; Zuo et al., 2018; Puhl et al., 2019). The approved drugs maraviroc and ibalizumab have the following mechanism of action: maraviroc is an antagonist of C chemokine receptor type 5, CCR5, and ibalizumab is a monoclonal antibody to the epitope on the CD4 receptor (Kinch and Patridge, 2014; Puhl et al., 2019). Enfuvirtide and albuvirtide block the fusion of viral and cellular membranes by binding to the HIV-1 gp41 subunit of the viral envelope that anchors the gp120 subunit, which normally binds to the CD4 receptor (Fung and Guo, 2004). Some other strategies for development of active molecules targeting HDFs are reported in the literature (Zhang et al., 2015; Tan et al., 2019).

Besides the creation of new inhibitors of HIV enzymes and HDFs, some other approaches are currently under development to treat HIV infection, namely the creation of anti-HIV vaccines (Hsu and O’Connell, 2017), immunotherapy with broadly neutralizing antibodies (Lacerda et al., 2013; Sok and Burton, 2018; Haynes et al., 2019), and creation of methods to cure latent HIV infection (Peterson and Kiem, 2018; Sadowski and Hashemi, 2019).

To date neither therapeutic nor preventive anti-HIV vaccine exists (Gray et al., 2016). Although hundreds of vaccine candidates have been clinically tested, only the RV144 trial has achieved positive yet moderate protection (Gao Y. et al., 2018). The difficulty in the creation of anti-HIV vaccine can be explained by the fact that people do not develop protective immunity to HIV infection; whereas almost all successful vaccines were created for diseases, for which the immunity can be developed after exposure to a live pathogen (Brett-Major et al., 2017). The development of effective anti-HIV vaccines gave rise to bioinformatics approaches for computational analysis of viral variants and corresponding host factors (i.e., T-cell epitopes) (Fischer et al., 2007; Korber et al., 2009; Barouch et al., 2013; Hulot et al., 2015; Khan et al., 2017), which can be beneficial for the creation of potential anti-HIV vaccine.

To create effective vaccine, deep understanding of interplay between HIV and human immune system is required. The HIV causes innate immune response and later adaptive T cytotoxic and humoral responses, which cannot completely cure infection due to several reasons (Levy, 2015). First, high mutation rate of virus causes formation of viral variants with antigens’ epitopes, which cannot be recognized by HIV-specific antibodies and CD8+ T cells (Gallo, 2015; Brett-Major et al., 2017). Second, HIV has additional mechanisms to escape from immune response (Dirk et al., 2016; Langer et al., 2019; van Stigt Thans et al., 2019). Third, in conditions of chronic infection, persistent exposure of T cells to high levels of antigen results in a severe T-cell dysfunctional state called exhaustion (Fenwick et al., 2019). Immune checkpoint molecules, including PD-1, CTLA-4, TIM-3, CD160, 2B4 and LAG-3, play a critical role in the maintenance of exhaustion and dysfunction. Administration of immune checkpoint inhibitors has therefore attracted considerable interest as a strategy to enhance HIV-specific T cell responses (Seddiki and Lévy, 2018; Mylvaganam et al., 2019). Fourth, since viral cDNA is integrated into the human genome, latent HIV reservoirs such as CD4+ T memory cells are present (Gallo, 2015; Pitman et al., 2018; Sadowski and Hashemi, 2019). These reservoirs do not produce viral particles, but they can give rise to infectious virions following activation by various stimuli, leading to viral rebound when cART is interrupted (Brett-Major et al., 2017). Fifth, mucous membranes are bottleneck for multiple viral variants, because only one (the so-called transmitted/founder variant) or a few HIV variants are able to overcome this barrier. These variants have unique properties allowing escaping from immune response in mucosa and transporting to lymph nodes, e.g., they are relatively more resistant to interferons and bind dendritic cells more efficiently (Iyer et al., 2017; Rios, 2018). Therefore the difficulty in developing effective preventive anti-HIV vaccine is to detect the viral variant, which is able to penetrate the mucosal barrier.

The development of therapeutic strategies to cure latent HIV infection is an important research direction along with the development of vaccines. Three main strategies are currently developed, namely gene therapy and genome editing of CCR5 or integrated HIV genome itself (Wang and Cannon, 2016; Peterson and Kiem, 2018), “shock and kill” and “block and lock” strategies (Gallo, 2016; Darcis et al., 2017; Pitman et al., 2018; Sadowski and Hashemi, 2019). “Shock and kill” approach is based on the application of latency reversing agents, which cause reactivation of viral gene transcription and generation of virions. Their application causes elimination of infected cells through immune response and virus-induced apoptosis; however, these processes are incapable of eliminating all infected cells, thus, additional methods are used together with the application of latency reversing agents (Pitman et al., 2018; Mylvaganam et al., 2019; Sadowski and Hashemi, 2019). “Block and lock” is an opposite strategy, which is based on prevention of viral RNA expression even in the case of T cell reactivation. It can be achieved by application of HIV Tat inhibitors, small interfering RNA (siRNA) or short hairpin RNA (shRNA) that can target and destroy the viral RNAs, and others (Darcis et al., 2017; Pitman et al., 2018; Sadowski and Hashemi, 2019). The more aggressive strategy is the application of gene therapy and genome editing. Several gene editing approaches including those based on Zinc Finger Nucleases (ZFN), transcription activator-like effector nucleases (TALEN) and Clustered Regularly Interspaced Short Palindromic Repeats/Cas-9 (CRISPR/Cas-9) have been applied to cause CCR5 disruption or remove/modify integrated HIV genome. These approaches demonstrated promising results for *in vitro* and animal experiments as well as in clinical trials (Wang and Cannon, 2016; Peterson and Kiem, 2018; Herrera-Carrillo et al., 2019) but they are still under investigation now (Vansant et al., 2020).

The studies of HIV-host interaction is important because it helps to understand the factors influencing the speed of disease progression and pathogenesis features at individual patients. It is known that some people, called long-term non-progressors, have the ability to suppress viremia to undetectable levels, while maintaining elevated CD4 cell counts in the absence of cART (Poropatich and Sullivan, 2011; Gonzalo-Gil et al., 2017). They are subdivided into several groups. For instance, elite controllers are HIV infected individuals, who suppress viremia to less than 50 copies/ml, while maintaining CD4 cell counts from 200 to 1000/ml. Viremic controllers achieve a lesser degree of virologic control (viral load between 200 to 2000 copies/ml), while usually maintaining CD4 cell counts less than 500/ml, in the absence of cART. The phenomenon of long-term non-progressors can be mainly explained by enhanced cellular immune response and decreased susceptibility of CD4+ T cells to HIV infection (Gonzalo-Gil et al., 2017). Investigation of mechanisms of HIV control in these groups of patients is very important, because mimicking similar responses in chronically infected individuals, e.g., by therapeutic vaccine, will lead to functional remission of HIV infection (Seddiki and Lévy, 2018).

Human CD4+ T cells are the most widely recognized and best-described cell type, which are infected by HIV; however, the virus can also replicate in cells of other types including monocytes and macrophages, and various kinds of dendritic and epithelial cells (Kandathil et al., 2016). In particular, HIV can infect the central nervous system’s various cells, such as microglia and astrocytes, which leads to neuroAIDS in about two-thirds of patients, and is characterized by a decline in brain function and movement skills (Elbirt et al., 2015; Clifford, 2017; Kumar et al., 2018; Olivier et al., 2018; Sillman et al., 2018). The corresponding condition is called HIV-associated Neurocognitive Disorder (HAND), which can be further categorized from asymptomatic HAND to HIV-associated dementia linked with cognitive impairment, motor dysfunction, speech problems, and behavioral changes. To date, the administration of cART allows significantly decrease the severity of HAND in HIV-infected subjects; however, low permeability of antiviral drugs through blood-brain barrier and presence of latent HIV infection considerably reduce the efficacy of therapy. To increase the concentration of anti-HIV compounds in the brain, various drug delivery approaches and prodrugs are developed (Kumar et al., 2018). Since HIV-1 acquires latency in various brain cells, e.g., perivascular macrophages, microglial cells, and astrocytes, the brain tissues represent an essential reservoir for HIV-1. In spite of cART administration, it can lead to chronic pathological implications because minimum viral genome transcription can continuously produce little virus, and viremia can be rebound upon latency reactivation (Van Lint et al., 2013); therefore, HAND treatment also requires the elimination of latent infection by various approaches described above (Marban et al., 2016; Proust et al., 2017).

Development of all above mentioned approaches require the deep understanding of the mechanisms of HIV-host interactions. To date, a large amount of these OMICs data about the HIV-host interaction has been recorded in the literature and in public databases. Network-based analysis allows for integrating OMICs data of various types, and it can be used to determine critical pathways and key proteins involved in the HIV life cycle, cellular and immune responses to infection, and different speed of disease progression. The identified proteins and pathways may represent targets for the development of new anti-HIV strategies as well as the optimization of the existing therapy.

This review consists of two parts. The first part contains a description of various types of OMICs data, such as HIV-human protein-protein interactions, RNA-RNA interactions, genomics, transcriptomics, proteomics, and epigenomics information as well as data from functional genomics screenings. We review the content and quantity of the corresponding HIV-related data from public resources, databases and literature. The second part contains a description of the network-based analysis of HIV-human interactions, including the types of networks, the methods of integrating them with the OMICs data, and methods of network topology analysis, which can be used to discover new HDFs and key cellular pathways that form the basis for developing new anti-HIV therapeutic and prophylactic strategies.

## Types of Omics Data Describing the HIV–Host Interaction

### Interactomics Data

#### Protein–Protein Interactions

The interaction between HIV and human proteins is the first event that causes subsequent changes in the cellular pathways and processes required for the viral life cycle. Thus, information on protein–protein interactions (PPIs) is the most common type of data, and it is used for the creation of network-based models of HIV infection (see below). The information on HIV-human PPIs can be extracted from at least six public databases that are focused on pathogen–host interactions (Table 1). The information in the specialized databases, in turn, was obtained from publications and more general PPI resources, e.g., BioGRID^1^, IntAct^2^, MINT^3^, DIP^4^, and STRING^5^. The proteins in these databases, except for the NCBI HIV-1 human interaction database^6^, are presented as SwissProt^7^ accession numbers, which have two primary features. First, they are gene-centric, so each identifier corresponds to a single gene. However, some HIV genes, such as gag, pol, and env, encode precursor polyproteins, which cleaved by HIV protease into two or more particular proteins (Kirchhoff, 2013). Second, each protein has several identifiers that correspond to different HIV groups, subtypes and even isolates, e.g., tat protein from HIV-1 group M subtype B refers to 20 SwissProt accession numbers corresponding to 20 different isolates. For illustrative purposes, we calculated the number of pairs listed as “HIV gene symbol – SwissProt identifier of human protein” for six databases, whereas the differences in PPIs between the HIV groups, subgroups, and isolates were ignored (Table 1).

**TABLE 1:** Public databases containing data on interactions between HIV and human proteins.

**Database**	**Number of HIV-1-human interactions**	**Number of human proteins interacting with HIV-1^1^**	**Number of HIV-2-human interactions**	**Number of human proteins interacting with HIV-2^1^**	**References**
NCBI database^2^	1037	842	–	–	Ako-Adjei et al., 2015
HPIDB	1668	1390	25	24	Ammari et al., 2016
PHISTO	1978	1460	27	26	Durmuş Tekir et al., 2013
VirHostNet	1077	985	13	13	Guirimand et al., 2015
Viruses.STRING	929	827	3	3	Cook et al., 2018
VirusMentha	1206	1052	14	13	Calderone et al., 2015
Total^3^	2910	2051	34	30	

*^1^The number of unique SwissProt identifiers that corresponds to human proteins interacting with HIV proteins. ^2^The database contains both direct and indirect functional interactions. We considered direct interactions based on the following keywords: “acetylated by,” “binds,” “cleaved by,” “cleaves,” “degraded by,” “dephosphorylated by,” “glycosylated by,” “interacts,” “is monomethylated,” “is polyubiquitinylated by,” “isomerized by,” “methylated by,” “modified by,” “myristoylated by,” “palmitoylated by,” “phosphorylated by,” “processed by,” “sulfated by,” “sumoylated by,” “ubiquitinated by.” ^3^Total numbers of unique interactions.*

**FIGURE 1:**
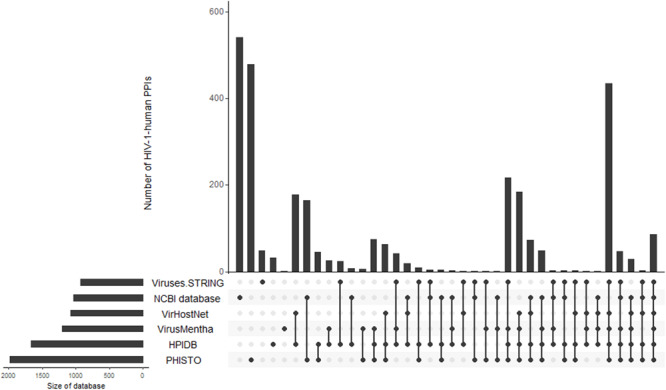
Intersections of HIV-1-human PPIs between six databases. *Y*-axis represents the numbers of HIV-1-human PPIs, which are either unique for a particular database or shared by two, three, four, five, and six databases. The connections between circles at the bottom part of figure represent intersections of PPIs between databases. The unconnected circles represent PPIs, which are unique for a particular database. The horizontal bars represent the total numbers of HIV-1-human PPIs in each database.

To demonstrate the intersections between the database contents, we created an Upset plot (Lex et al., 2014) (Figure 1). Figure 1 shows that the NCBI HIV-1 human interaction database and the PHISTO database^8^ have the highest numbers of unique HIV-1-human PPIs, with 540 and 475 respectively, whereas only 86 PPIs were present in all six databases. Among all the databases, the NCBI HIV-1 human interaction database was created using the manual curation of data taken from the literature. It contains a description of the functional significance of interactions, e.g., “phosphorylation,” “acetylation,” “activation,” and “inhibition.” In addition to direct interactions, the NCBI HIV-1 human interaction database also contains indirect ones, which primarily reflect an influence on the gene expression or protein activity (the corresponding indirect interactions are not shown in Table 1 and Figure 1).

By merging the data from six databases, we obtained 2910 unique HIV-1-human PPIs corresponding to 2051 individual human proteins (Table 1). The large number of human proteins interacting with HIV-1 can be explained by disadvantages in the experimental methods used to measure the PPIs (Goodacre et al., 2020). According to the largest database, PHISTO, the most common high-throughput approaches to identifying HIV-human PPIs are affinity-purification coupled to mass spectrometry, pull-down, and co-immunoprecipitation. These methods may address physical interactions in which two proteins are in the same complex but do not interact directly. According to some estimates, 50 to 70 percent of all PPIs may be physical but not direct (Goodacre et al., 2020). Significant numbers of PPIs obtained by high-throughput methods may be false positives (Goodacre et al., 2020). The VirHostNet^9^, VirusMentha^10^, and Viruses.STRING^11^ databases provide confidence scores that reflect the number of studies in which PPI was detected, and a number and quality of experimental techniques were used. The scores vary from 0 to 1 and allow for the selection of the most likely PPIs; however, the medians of the scores (0.21 for VirusMentha, 0.33 for VirHostNet, and 0.44 for Viruses.STRING) indicate that most of the interactions are low-confidence. Thus, the real number of direct interactions is potentially much lower than those presented in Table 1.

Since the PPI profiles for other viruses were shown to be different for different viral variants (Neveu et al., 2012; Goodacre et al., 2020), we calculated the numbers of interactions for particular groups and subtypes of HIV-1 by accounting for the viral isolates (Table 2). Almost all the data belong to HIV-1 group M, whereas the largest number of HIV 1-human PPIs and isolates is associated with subtype B, followed by subtypes A and D. The information about the groups and subtypes for the significant number of interactions (995 PPIs) is not presented in the databases. Merging of data on different groups and subtypes for the network analysis is not strictly correct, because the corresponding interaction patterns may be different (Neveu et al., 2012; Goodacre et al., 2020). Nevertheless, this finding is usually ignored, because the separate analysis of HIV-human PPI data is not possible for most of the groups and subtypes, except for group M subtype B, since they are associated with very few or no interactions (Table 2).

**TABLE 2:** Distribution of the numbers of PPIs between different HIV-1 groups and subtypes.

	**Number of isolates**	**Range of PPI numbers for different isolates^1^**	**Number of unique PPIs^2^**	**Databases^3^**
HIV-1 group M subtype A	3	162–350	353	b, d
HIV-1 group M subtype B	27	6–1875	1910	a, b, d, f
HIV-1 group M subtype C	1	5	5	b, d, f
HIV-1 group M subtype D	5	167–347	356	b, d, f
HIV-1 group M subtype F1	1	1	1	b, d
HIV-1 group M subtype G	1	1	1	b, d
HIV-1 group M subtype H	1	1	1	b, d
HIV-1 group M subtype U	1	139	139	d
HIV-1 group N	1	1	1	b, d
HIV-1 (group unknown)	–	995	995	a, b, c, d, e, f

*^1^Number of “SwissProt identifier of HIV gene – SwissProt identifier of human protein” pairs. ^2^Number of unique “HIV gene symbol – SwissProt identifier of human protein” pairs. Information about different isolates was merged. ^3^The data originated from the databases: (a) VirHostNet, (b) HPIDB, (c) NCBI database, (d) PHISTO, (e) Viruses.STRING, (f) VirusMentha.*

The available number of HIV-2-human interactions is lower than for HIV-1 (Table 1). One may suggest that this is observed because HIV-1 is studied more thoroughly than HIV-2 (Campbell-Yesufu and Gandhi, 2011).

The list of 2910 HIV-1-human PPIs is presented in Supplementary Table S1.

#### Interactions Between Messenger RNAs and Non-coding RNAs

There is growing evidence regarding the role of non-coding RNAs, such as micro RNA (miRNA) and long non-coding RNA (lncRNA), in HIV-human interactions (Lazar et al., 2016; Fruci et al., 2017; Balasubramaniam et al., 2018). Dozens of human miRNAs are known to bind the messenger RNAs (mRNAs) of HDFs and modulate HIV replication either positively or negatively. Human miRNAs can also provide anti-HIV activity through the direct targeting of the viral genome and viral mRNAs (Balasubramaniam et al., 2018). However, the HIV genome also encodes miRNA targeting human mRNA and miRNA as well as viral mRNAs. These interactions regulate apoptosis and cell survival, the events that give HIV-infected cells a survival advantage, and they have a positive impact on viral replication and provide an escape from the host immune response (Lazar et al., 2016; Fruci et al., 2017). Information about the interactions between HIV-1 and human RNAs can be obtained from three specialized databases, namely ViRBase^12^ (Li et al., 2015), VmiReg^13^ (Shao et al., 2015), and VIRmiRNA^14^ (Qureshi et al., 2014), which integrate the corresponding data from publications. The numbers of various types of interactions in the databases are shown in Table 3.

**TABLE 3:** Various types of interactions between HIV-1 and human RNAs presented in public databases.

**Type of interaction**	**ViRBase**	**VmiReg**	**VIRmiRNA**	**Total**
Human miRNA–viral mRNA	43	7	21	49
Human miRNA–human mRNA	50	–	3	51
Viral miRNA–human mRNA	21	13	2	21
Viral miRNA–viral mRNA	5	2	–	5
Viral miRNA–host miRNA	2	–	–	2

The ViRBase includes data on a few interactions between human lncRNAs and human or HIV mRNAs. For example, human lncRNAs 7SL and Y3 interact with members of the APOBEC3 family of human proteins, which possess antiviral activity through the deamination of viral RNA (Wang et al., 2007; Zhen et al., 2012). The lncRNA encoded by the NEAT1 human gene is involved in interactions with and posttranscriptional regulation of unspliced HIV-1 transcripts (Zhang et al., 2013).

The list of RNA-RNA interactions from the three databases is presented in Supplementary Table S2.

### Genomics, Transcriptomics, Proteomics, and Epigenomics Data

#### Genomics Data

The high mutation rate of the HIV genome is one of the reasons for the formation of multiple HIV variants, which have new antigen properties that allow the virus to escape from an immune response (Rogozin et al., 2005; Cuevas et al., 2015). However, polymorphisms in the human genome can also influence the susceptibility to and severity of HIV infections (Le Clerc et al., 2019; Tough and McLaren, 2019). The candidate gene studies revealed a 32-bp deletion in the CCR5 gene (CCR5-Δ32) encoding a co-receptor that is essential for HIV binding to CD4+ cells. The homozygotes of the CCR5-Δ32 mutation are resistant to HIV, whereas heterozygotes demonstrate slower disease progression. Other significant gene loci related to the viral load and HIV progression are located in HLA (human leukocyte antigen) genes. They encode the major histocompatibility complex, which presents peptides derived from HIV proteins to T-helpers, one of the critical stages of an immune response to infection. The genome-wide association studies (GWASs) allow for the linking of thousands and millions of genome polymorphisms, primarily single nucleotide polymorphisms (SNPs), to various phenotypes, e.g., the viral load, HIV susceptibility or disease progression (Le Clerc et al., 2019; Tough and McLaren, 2019). To date, more than 20 GWASs related to HIV were performed; however, they did not allow for the identification of new SNPs, which have strong, statistically significant and reproducible associations with HIV-associated phenotypes, such as viral load and capability to viremic control (Le Clerc et al., 2019). Given GWASs confirmed the significance of CCR5 and HLA gene polymorphisms, whereas newly revealed associations were weak and usually non-reproducible. Nevertheless, a few human genes may be considered as potential candidates, e.g., CXCR6 encoding C-X-C chemokine receptor type 6, which is one of the co-receptors for the binding of the HIV-2 and m-tropic HIV-1 strains along with CCR5. A detailed description of HIV-related GWASs explored to date is given in the review (Le Clerc et al., 2019). The corresponding associations between human genetic polymorphisms and HIV-related phenotypes can be obtained from the GWAS Catalog^15^, GWAS Central^16^ resources. The results of HIV-related genomics studies can also be accessed through the dbGaP database^17^.

#### Transcriptomics Data

Information on the expression levels of coding and non-coding RNAs is the most represented OMICs data type in HIV research. The public data from HIV-related transcriptomics experiments can be downloaded from Gene Expression Omnibus^18^ (GEO) and ArrayExpress^19^. We searched these databases with the keyword “HIV” and manually inspected the results. We found 232 transcriptomics experiments in which the mRNA, miRNA or lncRNA levels were measured by microarrays or RNA sequencing-based methods under different conditions, in different cell types, *in vivo* or *in vitro* (Table 4 and Supplementary Table S3). Most of the experiments were focused on measuring mRNA profiles, and, to a lesser degree, on miRNA profiles, whereas only one study was related to the lncRNA profile. Comparisons of RNA transcription profiles between CD4+ cells obtained from HIV-infected or healthy individuals were the most common types of experiments; however, additional conditions were taken into account in most experiments. For example, over 30 experiments were related to the comparison of transcriptional profiles between chronic progressors that are in a chronic stage of the infection and develop AIDS without using cART, viremic controllers, and elite controllers (see section “Introduction”). Most of the transcriptome measurements were performed on human cells derived from HIV-infected and healthy donors; however, in some cases, experiments were performed on human cell lines or primary cells infected with HIV *in vitro*. HIV infection of primary CD4 T cells requires T cell activation signals, and the activated cell is twice as likely as a non-activated cell to be productively infected with HIV-1 (Oswald-Richter et al., 2004; Biancotto et al., 2008). Therefore, primary CD4 T cells are activated artificially before the HIV-1 infection in studies *in vitro*; however, such stimulation may affect the cellular transcriptome, proteome, epigenome, and interactome, and introduce significant bias into results of the experiment.

**TABLE 4:** An overview of HIV-related transcriptomic experiments.

**Experiment type**	**Cell types**	**Conditions**
***in vivo (140)***	***Primary immune cells and tissues*:** peripheral blood mononuclear cells (29), CD4+ T cells (24), whole blood (19), CD8+ T cells (13), monocytes (12), B cells (4), NK cells (3), lymph nodes (2), plasma (2), and others ***Other primary cells and tissues*:** frontal cortex (6), jejunum, colon or rectal cells (4), lymphoma cells (3), adipose cells (2), retinal cells (2), skeletal muscle cells (2), and others	***HIV infection status:*** infected, uninfected (65)
		***HIV progression types:*** resistant to HIV, long-term non-progressors, elite controllers, chronic progressors, and others (36)
		***HIV infection stages*:** acute, chronic (latent), AIDS (4)
		***Quantitative characteristics of HIV infection:*** CD4+ cells count and HIV RNA level (5)
		***HIV associated diseases*:** HIV-associated neurocognitive disorders, HIV-associated tumors, and others (22)
		***HIV co-infections*:** mycobacterium tuberculosis, *Neisseria gonorrhoeae*, pneumococcal meningitis, hepatitis C virus, kaposi’s sarcoma-associated herpesvirus, and others (17)
		***Small molecule treatment including antiretroviral drugs*:** treated or not treated patients, before or after therapy, before and after treatment interruption, and other (23)
		***Vaccine trials (6)***
		***Microbiome changes*:** transcriptional changes in gut, tongue and lung microbiome induced by HIV (3)
		***Cell subtypes:*** comparison of transcription profiles between immune cell subtypes (9)
***In vitro (93)***	***Primary cells*:** CD4+ T cells (15), monocyte-derived macrophages (15), peripheral blood mononuclear cells (8), monocyte-derived dendritic cells (6), macrophages (2), primary human neurons (2), and others ***Cell lines:*** Jurkat cells (12), SupT1 cells (7), HEK293T cells (4), CEM-SS cells (3), ACH-2 and A3.01 cells (2), SH-SY5Y cells (2), U1 cells (2), U-937 cells (2), WE17/10 cells (2), and others	***HIV infection status:*** infected, uninfected (51)
		***HIV infection time-series*:** different times after infection *in vitro* (22)
		***HIV latency*:** viral latency in various CD4+ cells and reactivation by different agents (9)
		***Treatment of cells with small molecule substances including antiretroviral drugs (11)***
		***Treatment of HIV-infected cells with cytokines (4)***
		***Infection of cells with various HIV variants and vectors expressing wild type or mutated HIV proteins (30)***

*The numbers of corresponding experiments calculated as numbers of GEO and ArrayExpress identifiers are given in brackets.*

In addition to the investigation of human cell transcriptome, three experiments were focused on the transcriptomes of the human intestinal, lung, and oral microbiota from HIV-infected and healthy subjects (see Table 4), because they seem to play an essential role in HIV progression and the development of opportunistic infections (Dang et al., 2012; Vujkovic-Cvijin et al., 2013; Iwai et al., 2014).

We found plenty of experiments in which microarrays and “bulk” RNA sequencing were used to measure the transcriptome; however, the investigated cell populations may really be heterogenic, and measurement of “mean” transcript expression values can introduce significant bias into results of the analysis. For instance, the proportion of viral-infected cells is quite low even in the CD4+ T cell population of HIV-1 infected patients (Baxter et al., 2016); therefore, the results of “bulk” studies do not necessarily reflect the characteristics of viral-infected cells themselves. To overcome this limitation, some studies (Sedaghat et al., 2008; Vigneault et al., 2011; Cohn et al., 2018; Chen et al., 2019) used methods allowing purifying the cell subpopulations, such as latently infected, activated cells and cells with HLA-DR^–^ phenotype, before measuring the transcriptome. These methods can be coupled with single-cell RNA sequencing, which allows for the identification of clusters of blood cells or cell subtypes with different transcriptional responses to HIV infection (Chen et al., 2019; Kulkarni et al., 2019). For example, Cohn and colleagues developed a method to enrich and isolate reactivated latent cells by combining antibody staining, magnetic enrichment, and flow cytometry, accompanied by single-cell RNA sequencing. They found that reactivated latent cells produce full-length viruses which are identical to those found in viral outgrowth cultures, and represent clones of *in vivo* expanded T cells as determined by the sequence of their T cell receptors. Gene expression analysis revealed that these cells share a transcriptional profile that includes expression of genes implicated in silencing the virus and allows for cell division without activation of the cell death pathways (Cohn et al., 2018).

Additionally, we found six HIV-related studies in the GEO database for which single-cell RNA sequencing was applied (Supplementary Table S4). For example, Farhadian et al. (2018) performed single-cell RNA sequencing on cerebrospinal fluid and blood from adults with and without HIV. They found a rare (<5% of cells) subset of myeloid cells that are found only in cerebrospinal fluid and present a gene expression signature that overlaps significantly with neurodegenerative disease-associated microglia. This immune cell subset may perpetuate neuronal injury during HIV infection (Farhadian et al., 2018).

Most of the HIV-related experiments were performed to identify differentially expressed genes (DEGs) between two or more conditions with subsequent functional annotation. The functional annotation of the DEGs is usually performed by pathway enrichment analysis (Khatri et al., 2012; Kuleshov et al., 2016), which allows for the identification of pathways, Gene Ontology^20^ biological processes or other functional categories that were “enriched” by DEGs compared to the background gene set, e.g., all human genes. For example, Devadas et al. (2016) identified the transcriptional changes in the peripheral blood mononuclear cells during HIV-1 and HIV-2 infection. HIV-1 caused changes in the expression of 316 genes, whereas HIV-2 changed the expression of only 57 genes. The pathway enrichment analysis allowed for the identification of the pathways and Gene Ontology biological processes associated with the infection. The authors found that the pathways and cellular processes perturbed by HIV-1 and HIV-2 are not the same, e.g., only the HIV-1 virus influenced genes related to the cell cycle and apoptosis. The observed differences possibly explain the different rates of disease progression from HIV-1 and HIV-2 and the observations showed that HIV-2 is generally less pathogenic than HIV-1 (Devadas et al., 2016).

It should be noted that some transcriptomics studies were performed to measure the gene expression changes under the application of anti-HIV vaccine candidates (Zak et al., 2012; Fourati et al., 2019). Currently, there are no effective anti-HIV vaccines; thus, understanding the cellular mechanisms of the immune response at the transcriptome level to more or less effective vaccine candidates, and in individuals with stronger or weaker vaccine effect may help researchers to develop more effective ones (Haynes et al., 2016; Trovato et al., 2018).

Nine transcriptomics studies are related to another important problem: HIV latency. The comparison of transcriptional profiles of latently and productively infected cells, as well as cells treated with different latency reversing agents may allow revealing mechanisms of latency and identifying new more effective solutions for “shock and kill” or “block and lock” approaches. For example, White et al. (2016) compared transcription profiles of uninfected and latently infected central memory cells, and identified 826 DEGs, many of which were related to p53 signaling. The authors found that inhibition of the transcriptional activity of p53 during HIV-1 infection reduced the ability of HIV-1 to be reactivated from its latent state. Their observations may help to develop new latency reversing agents (White et al., 2016).

In addition to the differential expression analysis, transcriptomics data may be used for the construction of co-expression and gene regulatory networks (see below).

Beside GEO and ArrayExpress resources, specialized HIVed database^21^ also provides access to some HIV-related transcriptomics and proteomics experiments derived from literature. It contains data on gene expression levels during HIV infection and replication as well as at HIV latency (Li et al., 2017).

The list of HIV-related transcriptomics experiments prepared in this study is presented in Supplementary Table S3.

#### Proteomics Data

The mass spectrometry-based proteomics approaches allow researchers to measure the absolute or relative amounts of proteins in human cells under various conditions. The public proteomics data describing HIV–human interaction can be obtained from ProteomeXChange^22^, which contains information from the PRIDE Archive^23^ and some other sources. Several datasets are also published in GEO. We manually inspected the corresponding databases and performed a search in PubMed using the keywords “HIV” and “proteome.” We found more than 900 publications and selected those 25 proteomics studies, where genome-wide protein profile was measured, and corresponding data was made publicly available (see Supplementary Table S5). Most of these studies are focused on identifying differentially expressed proteins (DEPs) between the same conditions as those in the transcriptomics studies (see above); however, some of the studies have features unique to proteomics experiments (see below). The transcript and protein levels have only weak correlation across different experimental conditions, mainly because of the existence of complex regulation at all stages of gene expression: from transcription to regulation of protein translation, maturation, transport and degradation (Vogel and Marcotte, 2012; Wang et al., 2019). The changes in protein level may also not be accompanied by changes in transcript level and vice versa. For example, HIV protease destroys the human proteins, which leads to a decrease in protein levels, but may not lead to the changes in the corresponding transcripts. Thus, the simultaneous assessment of changes in the transcriptome and proteome under the same cells and other experimental conditions is extremely important for the building of *in silico* integrative models that provide the most accurate results. For example, Golumbeanu et al. (2019) measured both the proteomics and transcriptomics responses to HIV-1 infection in SupT1 CD4+ T cells at five time points. They identified new HDFs involved in a wide range of cellular processes, such as cell signaling, immune response, cell cycle, gene expression, or metabolism. The majority of these factors were not found differentially expressed at the RNA level. Unfortunately, other published studies were only focused on the changes in the proteome.

Another advantage of proteomics approaches is the capability to measure the changes in post-translational modifications of human proteins during HIV infection. We found three studies related to the estimation of such changes (Greenwood et al., 2016; Yang et al., 2016; Lapek et al., 2017). Greenwood et al. (2016) performed an analysis of more than 6500 HIV and cellular proteins in the CEM-T4 cell line infected by wild type and Vif-deficient viruses. Among others, they measured changes in phosphoproteome and found Vif-dependent hyperphosphorylation in more than 200 cellular proteins, particularly the substrates of the aurora kinases (Greenwood et al., 2016). Since the distinct profile of glycosylated surface proteins can be used for targeting latently infected cells, Yang et al. (2016) measured differences in the glycol-proteome in ACH-2 and A3.01 cell lines, which are models of latently infected and uninfected cells. They identified a change in the levels of 236 glycosite-containing peptides from 172 glycoproteins between two cell lines. These proteins participate in cell adhesion, immune response, glycoprotein metabolism, cell motion, and cell activation (Yang et al., 2016). The study by Zheng et al. (2011) aims at the identification of differences between immune response to different opportunistic infections (Epstein-Barr virus and Kaposi’s Sarcoma) based on proteome analysis.

Most proteomics experiments were performed by mass spectrometry-based approaches; however, a few studies used protein arrays, which are usually focused on particular sets of proteins. For example, Yang et al. (2013) aimed at the identification of autoantigens recognized by the broadly neutralizing antibodies to 2F5 and 4E10 epitopes of HIV-1 gp41. The corresponding protein array contained more than 9400 recombinant human proteins. As a result, the authors found human kynureninase and splicing factor 3b subunit 3 acting as human self-antigens.

The list of HIV-related proteomic experiments is presented in Supplementary Table S5.

#### Epigenomics Data

HIV-related epigenomics studies are focused on the changes in DNA methylation profiles (Zhang et al., 2016, 2018; Liu et al., 2019), post-translational modifications of histones (Marban et al., 2011; Park et al., 2014; Lucic et al., 2019), genome-wide chromatin accessibility (Johnson et al., 2018), and the identification of DNA binding/occupancy sites for human/HIV proteins (Marban et al., 2011) based on ChIP-chip, ChIP-seq, and ATAC-seq approaches (Supplementary Table S6). The corresponding data can be obtained through the GEO database. In total, we found 34 studies related to changes in the epigenome under HIV infection. Importantly, some of them have linked transcriptomics data, which was obtained during the same experiments. The list of HIV-related epigenomics experiments is presented in Supplementary Table S6, whereas some examples are given below.

Marban et al. (2011) searched the HIV-1 Tat protein binding sites in the human genome using the ChIP-seq approach. In parallel, they performed the corresponding transcriptomic study as well as ChIP-chip experiments to identify histone H3 acetylation sites. All the measurements were taken in Jurkat-Tat and Jurkat cells. The authors found that only ∼7% of the Tat-bound regions are near transcription start sites at gene promoters, whereas ∼53% of the Tat target regions are within DNA repeat elements. They also revealed that Tat binding sites are not significantly associated with DEG promoters, whereas changes in histone H3 lysine 9 acetylation are significantly associated (Marban et al., 2011).

Some of the epigenomics studies were focused on the investigation of HIV-1 integration sites. It is known, that viral DNA integration is not a random process, but HIV-1 predominantly integrates into open chromatin regions of active transcription, including genes that were activated in cells after infection by HIV-1 (Schröder et al., 2002; Lucic et al., 2019). Lucic et al. (2019) revealed that targeted genes are proximal to super-enhancer genomic elements and cluster in specific spatial compartments of the T cell nucleus. They showed that these gene clusters acquired their location during the activation of T cells and concluded that the clustering of these genes, along with their transcriptional activity, are the major determinants of HIV-1 integration in T cells (Lucic et al., 2019). Demeulemeester et al. (2014) identified the amino acid residues in HIV-1 integrase that directly contact target DNA bases and affect local integration site sequence selection. They found natural polymorphisms, which retarget viral integration away from gene dense regions. These variants were associated with rapid disease progression in patients with a chronic HIV-1 subtype C infection.

The epigenetic mechanisms, including histone modifications, may affect the transcriptional silencing of HIV and viral latency. Park et al. (2014) performed a genome-wide analysis of histone modifications in HIV-1 latency cell lines using ChIP-seq. They revealed that HIV-1 latency led to the downregulation of histone H3K4me3 and H3K9ac levels in 387 and 493 regions and upregulation in 451 and 962 sites. The genes associated with up- and down-regulated histone levels participate in various pathways and processes: cell death, protein import into nucleus, T cell activation, cell cycle, cell proliferation, and metabolic process. Authors also compared obtained results with the data on gene expression and found that the cell cycle regulatory genes such as CDKN1A and cyclin D2 identified by differentially modified histones might play an essential role in maintaining the HIV-1 latency (Park et al., 2014).

#### Data on Functional Genomics Screenings

To identify the HDFs, a genome-wide inactivation of gene expression through small interfering RNA (siRNA) and small hairpin RNA (shRNA) can be performed. In three large-scale studies conducted in 2008, 842 human genes were identified as HDFs (Brass et al., 2008; König et al., 2008; Zhou et al., 2008). Brass A. L., König R., and Zhou H. along with their colleagues identified 273, 295 and 230 genes as potential HDFs using corresponding siRNAs transfected into HeLa-derived TZM-bl, HEK-293 and HeLa P4-R5 cell lines; however, the percentages of shared genes between the studies were minimal, ranging from 3 to 6% (Bushman et al., 2009). A year later, Yeung et al. (2009) performed the corresponding screening on the Jurkat cell line using shRNAs and identified 252 potential HDFs, which also have minimal overlap with genes from three previous studies (Yeung et al., 2009). These results can be explained by differences in the cell lines or type of reporter, experimental noise, and differences between time points and filtering thresholds (Bushman et al., 2009; Tough and McLaren, 2019). Bushman et al. (2009) performed a Gene Ontology enrichment analysis for genes from three first screening studies and found that the overlaps between the identified cellular processes are higher than they are at the individual gene level. Thus, the revealed potential HDFs may represent at least a basis for further research with more accurate methods and primary human CD4+ cells.

Recently, to identify potential HDFs, Park et al. (2017) performed genome-wide knockout screening using CRISPR-Cas9 lentiviral single-guide RNA constructs, which have higher sensitivity and specificity than screens based on RNA interference. Their research was conducted on GXRCas9 cells and allowed for the identification of 5 HDFs, with HIV co-receptors CD4 and CCR5, which are well-known targets of the anti-HIV drugs maraviroc and ibalizumab (Kinch and Patridge, 2014; Puhl et al., 2019) as well as TPST2, SLC35B2 and ALCAM, which are new potential targets. Genes TPST2 and SLC35B2 encode tyrosylprotein sulfotransferase 2 and solute carrier family 35 member B2, functioning in the same pathway as sulfate CCR5, which facilitates its recognition by the HIV envelope. The ALCAM gene encodes activated leukocyte cell adhesion molecule-mediating cell aggregation, which is required for cell-to-cell HIV transmission. The results were validated in primary human CD4+ T cells through a Cas9-mediated knockout and an antibody blockade. These three human proteins can be used in further research as new potential anti-HIV targets.

## Network-Based Integration and Analysis of Omics Data Describing HIV–Host Interaction

**FIGURE 2:**
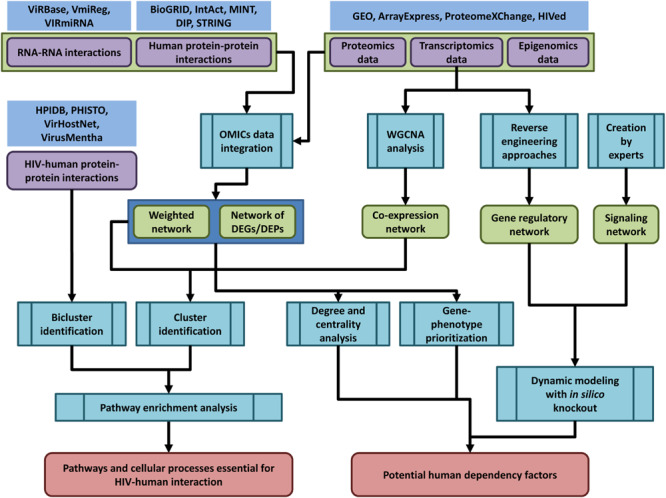
The general pipeline of network-based analysis of HIV-related OMICs data. Public databases provide access to OMICs data on human and HIV-human protein-protein interactions (HPIDB, PHISTO, VirHostNet, VirusMentha, and others, see Table 1 and Supplementary Table S1), interactions between human and viral coding/non-coding RNAs (ViRBase, VmiReg, VIRmiRNA databases, Table 3 and Supplementary Table S2), transcriptomics, proteomics and epigenomics data (GEO, ArrayExpress, ProteomeXChange, HIVed databases, Supplementary Tables S3–S6) (nodes of purple color in the figure). The HIV-related OMICs data can be used to create context-specific protein-protein interaction networks, co-expression, gene regulatory and signaling networks (nodes of green color in the figure). The context-specific networks can be constructed by weighting protein–protein interactions using transcriptomics, proteomics or epigenomics data, or by taking into account only DEG/proteins (DEGs/DEPs). Co-expression networks can be created using transcriptomics data and weighted gene correlation network analysis (WGCNA). Gene regulatory networks can be inferred from transcriptomics data using reverse engineering approaches, whereas signaling networks are usually manually created by experts based on a great deal of information regarding the protein interactions, post-translational modifications, and other data types. The created networks can be used for different types of analysis (nodes of blue color in the figure): (1) identification of dense communities in human protein-protein interaction and co-expression networks (clusters or modules), or in HIV-human interaction networks (biclusters). The pathway enrichment analysis applied to clusters and biclusters allows identifying pathways and cellular processes, which are essential for HIV-human interaction; (2) degree and centrality analysis, gene phenotype prioritization analysis, as well as dynamic modeling with *in silico* gene knockout allows identifying proteins, which are the most essential for HIV-human interaction (host dependency factors), and can be considered as potential targets for new anti-HIV therapeutic approaches.

The general pipeline of network-based analysis of HIV-related OMICs data is shown in the Figure 2.

### Protein–Protein Interaction Network-Based Analysis

#### Creation of Protein–Protein Interaction Networks

Protein–protein interaction networks (PPI networks) are the most common type of networks used in HIV-related studies. This is due to the high amount and availability of interaction data between human proteins (Csermely et al., 2013; Miryala et al., 2018) as well as human and HIV proteins (see Table 1 and Supplementary Table S1). The nodes of the PPI network are proteins, whereas the edges represent the direct or physical interactions between them, which are usually obtained by high-throughput *in vitro* experiments (Gillen and Nita-Lazar, 2019). The network may contain only one type of node representing human proteins, or two types of nodes representing both human and HIV proteins. Since PPI networks are derived from *in vitro* experiments, they contain proteins, which are not expressed in CD4+ cells and all the possible interactions that may take place in all the tissues, cell types, and conditions. To create context-specific networks representing molecular interactions under specific HIV-related conditions, the integration of the PPIs with other types of OMICs data is required (Figure 2). This integration can be performed in at least three ways. First, all the interactions can be measured in specific cells, under particular conditions, e.g., HIV-infected CD4+ T cells (Luo et al., 2016); however, due to their high cost, this type of interactomics experiment is not usually performed. Second, subnetworks containing only human proteins essential for the HIV life cycle can be created. Human proteins encoded by DEGs, immediate DEPs, HDFs derived from functional genomic screens or human proteins that are physically interacting with HIV can be used for this purpose (van Dijk et al., 2010; Xu et al., 2014; Shityakov et al., 2015; Gao L. et al., 2018; Saha et al., 2018). Third, the context-specific PPI network can be created from a global network by eliminating proteins that are not expressed under particular experimental conditions (Kotlyar et al., 2016; Basha et al., 2017), or by changing the edge weights using transcriptomics, proteomics and epigenomics data (Chen and Li, 2016; Li and Chen, 2018; Csösz et al., 2019). In addition to pure PPI networks, which contain only human/HIV proteins, integrated networks can be created. These networks include various types of nodes representing proteins, mRNAs, non-coding RNAs, or genes, and help in obtaining more accurate results than when only PPIs are studied (Chen and Li, 2016; Li and Chen, 2018). For example, Li and Chen (2018) created HIV-1-human interspecies protein–protein and miRNA interaction networks for different stages of HIV infection in CD4+ T cells, with reverse transcription, integration/replication, and the late stages of the HIV life cycle. The networks differed by edge weights, which were calculated using the corresponding transcriptomics data and stochastic dynamic modeling (Li and Chen, 2018).

The purposes of analyzing HIV-related PPI networks include the identification of human proteins essential for the HIV life cycle based on the topological characteristics of the networks, the identification of dense communities of proteins in the network, which may perform similar functions in the cell, and predictions of new potential HDFs by gene-phenotype prioritization algorithms.

#### Identification of Essential Proteins in a Protein–Protein Interaction Network

**FIGURE 3:**
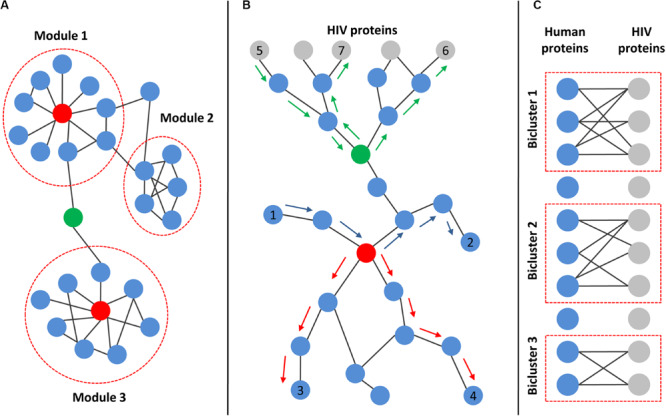
Main topological characteristics of human and HIV–human protein–protein interaction networks. **(A)** Example of a network with modular structures. Modules are dense communities of nodes that are highly interconnected but weakly connected to other nodes in the network. Red nodes represent “hubs,” which are proteins with a high number of interactions. Green node is an example of “bottleneck,” which exclusively connects distinct modules. **(B)** Principle of centralities calculation. Both closeness and betweenness centralities rely on the shortest paths between pairs of nodes in the network. The closeness centrality is the average length of the shortest paths between the protein and all the other proteins in the network (the shortest paths between red-colored node and nodes 3 and 4 are marked by red arrows). The betweenness centrality quantifies the number of times a protein acts as a bridge along the shortest path between two other proteins in the network (the shortest path between node 1 and node 2 passing through the red-colored node is marked by blue arrows). If the network includes both interactions between human proteins (blue nodes) as well as interactions between human and HIV proteins (gray nodes), then the modified betweenness centrality can be calculated. It reflects the number of times a human protein acts as a bridge along the shortest path between two HIV proteins (the shortest paths between node 5 and node 6 or node 7 passing through green-colored node are marked by green arrows). **(C)** Illustration of bipartite graph and biclusters. The bipartite graph contains two types of nodes (corresponding to HIV and human proteins) and edges connecting only nodes of different types, but not the same type. The corresponding data can be used to identify biclusters, which contain human proteins that interact with a common set of HIV proteins.

The degree of protein found in a network is simply the number of direct interactions with other proteins. The distribution of degree in real PPI networks follows power low, so most proteins have a low number of interactions, but a small number of proteins have a high number of interactions (Csermely et al., 2013; Miryala et al., 2018). The last type of proteins is called “hubs,” and they are critical for cell viability (see Figure 3A). The disturbance of their function can lead to cell death or carcinogenesis (Csermely et al., 2013; Miryala et al., 2018). In addition to “hubs,” the PPI networks contain proteins, called “bottlenecks,” which have few interactions but exclusively connect distinct modules and are therefore critical for cell survival (Figure 3A). These proteins have high centrality measures (Csermely et al., 2013; Miryala et al., 2018; Li et al., 2020). Two types of centrality are usually used, closeness centrality and betweenness centrality. The closeness centrality is the average length of the shortest paths between the protein and all the other proteins in the network. The betweenness centrality quantifies the number of times a protein acts as a bridge along the shortest path between two other proteins in the network (Figure 3B). The degree and centrality measures can be used to identify the essential human proteins influencing HIV–human interactions (Dickerson et al., 2010; van Dijk et al., 2010; Huang et al., 2011; Li et al., 2013; Ma et al., 2013; Bandyopadhyay et al., 2015; Xie et al., 2015). For example, Huang et al. (2011) compared gene expression profiles from CD4+ T cells between HIV-1-resistant and susceptible subjects using Minimum Redundancy-Maximum Relevance and Incremental Feature Selection algorithms. They identified 185 genes for which the expression levels distinguished between HIV-resistant and susceptible individuals with 85.2% accuracy. The authors identified 29 proteins from the 185 total based on the calculation of modified betweenness centrality in the HIV-1-human PPI network. This network included both interactions between human proteins as well as interactions between human and HIV-1 proteins. The modified betweenness centrality reflects the number of times a human protein acts as a bridge along the shortest path between two HIV-1 proteins (Figure 3B). Twenty-nine identified human proteins may be considered as targets for the disruption of communication between virus-targeted proteins and the prevention of viral infection (Huang et al., 2011). Shityakov et al. (2015) compared transcription profiles in frontal cortex between samples from AIDS patients with and without apparent features of HIV-associated encephalitis and dementia. They identified 1528 DEGs, which are mainly involved in the immune response, regulation of cell proliferation, cellular response to inflammation, signal transduction, and viral replication cycle. The authors created human PPI network containing only proteins encoded by DEGs, and identified hubs, e.g., heat-shock protein alpha, class A member 1, and fibronectin 1, which seem to play essential roles in the pathogenesis of HIV-associated encephalitis (Shityakov et al., 2015).

#### Identification of Clusters (Modules) and Biclusters in a Protein–Protein Interaction Network

At the higher level of network organization, modular structures can be found. The network module (cluster) is a dense community of nodes, which are highly interconnected with one another but weakly connected to other nodes in the network (Csermely et al., 2013; Miryala et al., 2018; Wu et al., 2019) (see Figure 3A). The proteins from the module tend to perform the same biological functions, e.g., a module may represent the dense part of the signaling pathway or complex cellular machinery such as one related to the DNA polymerase protein complex. The identification of modules in human PPI networks integrated with HIV-related OMICs data with subsequent functional annotation using pathway enrichment analysis allows researchers to reveal the particular mechanisms of HIV-host interactions (Xu et al., 2014; Amberkar and Kaderali, 2015; Yang et al., 2019). For example, Amberkar and Kaderali (2015) identified modules in the human PPI network using the ClusterONE algorithm and found that they are enriched by potential HDFs and HRFs from functional genomic screens (see above). Modules were then filtered based on network topology and semantic similarity measures, and the remaining two modules were finally interpreted for their biological significance using Gene Ontology and pathway enrichment analysis. As a result, the authors revealed that proteins from the first module were involved in gene transcription, whereas proteins from the second module participated in mRNA processing and splicing. Interestingly, the transcriptional regulation was not revealed in enrichment analyses of the individual screens, which may indicate the importance of module identification before enrichment because the inclusion of protein neighborhoods from corresponding clusters in the analysis may increase its power and sensitivity (Amberkar and Kaderali, 2015).

Interactions between human and HIV proteins can be presented as a bipartite graph, which contains two types of nodes (corresponding to HIV and human proteins) and edges connecting only nodes of different types, but not the same type (see Figure 3C). The corresponding data can be used to identify biclusters (Figure 3C), containing human proteins that interact with a common set of HIV proteins (MacPherson et al., 2010; Maulik et al., 2011, 2013). These topological structures are called “biclusters” because they contain two types of nodes, human and HIV proteins. The human proteins from biclusters represent the gateway proteins that are most affected by the HIV infection. Maulik et al. (2011) identified 14 overlapping biclusters using the original algorithm and bipartite network with two types of nodes corresponding to human and HIV-1 proteins. These biclusters formed a strongly connected subnetwork containing 7 HIV-1 and 19 human proteins. The list of corresponding human proteins is enriched with kinases and other proteins from signaling pathways (Maulik et al., 2011).

#### Gene-Phenotype Prioritization Algorithms for Identifying Potential HDFs

The proteins from the dense clusters in the PPI network tend to perform the same biological functions. This finding underlies many gene-phenotype prioritization algorithms, which are used to predict gene participation in cellular processes as well as to predict new associations between genes and human diseases (Lan et al., 2015; Fiscon et al., 2018). The corresponding methods can be divided into the following two groups: (1) local methods, which are based on the search for direct interactions between candidate genes and genes with a known phenotype; and (2) global methods, which model how the information flow in the PPI network is used to assess the proximity and connectivity between genes with the established phenotype and candidate genes. The general principle of algorithms is “the closer candidate genes to known phenotype genes in the network, e.g., they are in the same module, the higher score of the algorithm will be obtained.” Gene-phenotype prioritization algorithms and PPI networks were used to predict new HDFs essential for HIV-host interaction (Murali et al., 2011; Emig-Agius et al., 2014). For example, Murali T. M. used the original SinkSource algorithm and human PPI network to predict new potential HDFs that influenced HIV–host interactions (Murali et al., 2011). They used 545 genes from the three earliest genomic functional screens (Brass et al., 2008; König et al., 2008; Zhou et al., 2008) (see above) that were present in the PPI network as known HDFs. The Gene Ontology enrichment analysis, which was performed for the top 1000 predicted proteins, revealed that the obtained cellular processes are HIV-related, e.g., in RNA splicing, translation initiation, oxidative phosphorylation, and others. The authors also found that a significant number of the obtained potential HDFs interacted with HIV proteins. The predicted HDFs, along with those derived from genomic screens, may represent potential pharmacological targets for treating HIV infections.

### Co-expression Network-Based Analysis

**FIGURE 4:**
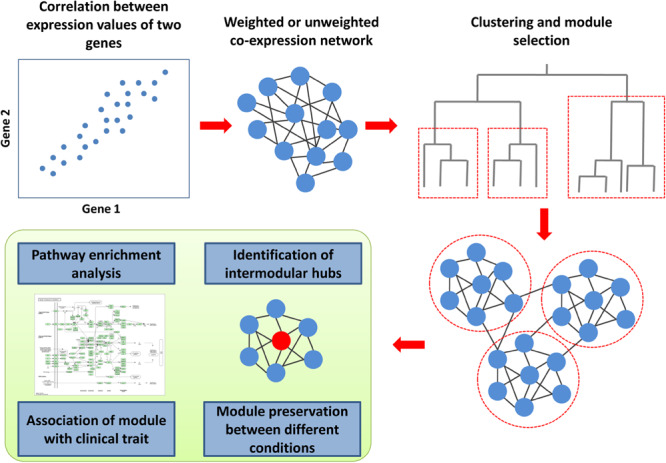
Co-expression analysis framework. To determine co-expression, the correlation between expression values for all gene pairs must be calculated. The weighted or unweighted co-expression networks can be created based on these values. A cluster analysis, which is based on co-expression values, allows identifying modules (clusters) of co-expressed genes. The co-expression module contains genes that could be regulated by the same transcription factors and have similar biological functions. The genes from each module can be used for pathway and Gene Ontology enrichment analysis to identify corresponding biological functions. Weighted gene correlation network analysis (WGCNA) is the most popular framework for the creation of co-expression networks. Along with the identification of modules, it can be used to estimate module preservation between two networks created for different conditions, reveal modules associated with a clinical trait of interest and find intermodular “hubs,” which could be the essential genes regulating the expression of the other genes in the module.

Co-expression is the simultaneous expression of two or more genes so that the transcription of two genes changed similarly under different conditions (Figure 4). The co-expression of two genes may reflect the transcription regulation by the same transcription factors. To determine co-expression, various measures can be employed, e.g., Pearson or Spearman correlation coefficients, mutual information, or Euclidean distance (van Dam et al., 2018). The unweighted co-expression network can be constructed by selecting a threshold on a co-expression measure so that the edge between the two genes exists when the corresponding value is higher than the threshold. The weighted co-expression networks contain all the possible edges between all the genes for which the weights are calculated using several functions from co-expression measures. A co-expression network can be created for particular cell types and conditions using microarray or RNA sequencing-based data (van Dam et al., 2018). The most common type of co-expression analysis is related to the identification of network modules (Figure 4). The co-expression module contains genes that could be regulated by the same transcription factors and have similar biological functions as in modules from the PPI networks. The most popular framework for the creation of co-expression networks and the analysis of modules is the weighted gene correlation network analysis (WGCNA) (Zhang and Horvath, 2005; Langfelder and Horvath, 2008; van Dam et al., 2018). The WGCNA can be used to create co-expression networks, identify modules, estimate module preservation between two networks created for different conditions, reveal modules associated with a clinical trait of interest and find intermodular “hubs,” which could be the essential genes regulating the expression of the other genes in the module. To date, several studies have been focused on the creation and analysis of co-expression networks for HIV-related conditions (Ma et al., 2011; Levine et al., 2013a, b; Xu et al., 2013; Ray and Bandyopadhyay, 2016; Mosaddek Hossain et al., 2017; Ray and Maulik, 2017; Quach et al., 2018; Ding et al., 2019; Nguyen et al., 2019). Most of them were based on transcriptomics data from CD4+ and CD8+ cells, which reflect different stages of HIV infection or progression types, e.g., chronic progressors, viremic controllers, elite controllers or subjects who were utterly resistant to the virus (Ma et al., 2011; Xu et al., 2013; Ray and Bandyopadhyay, 2016; Mosaddek Hossain et al., 2017; Ray and Maulik, 2017; Ding et al., 2019). Some other studies were focused on conditions related to HAND (Levine et al., 2013a, b; Quach et al., 2018). Almost all of the studies applied WGCNA to identify co-expression modules, which are preserved between different conditions, or modules related to a particular state, with subsequent Gene Ontology and pathway enrichment analysis of the modular genes.

Ray and Bandyopadhyay (2016) created a co-expression network for the acute phase of HIV infection with the subsequent identification of modules using WGCNA algorithm and transcription data from CD4+ and CD8+ T cells. The analysis on the preservation of modules across chronic and non-progressor stages was performed using the original rank aggregation algorithm, which is based on the comparison of module ranks and determined from multiple module characteristics, between HIV stages. The authors found 30 modules in the network for the acute phase of HIV infection, but not all of them were preserved in other stages. The genes from modules, according to the pathway enrichment analysis, participate in processes related to the immune system, the regulation of transcription, RNA processing, splicing and translation, cell cycle and apoptosis, and energy derivation as well as cytoskeleton regulation. The authors also performed a transcription factor enrichment analysis and found some novel factors such as “FOXO1,” “GATA3,” “GFI1,” “IRF1,” “IRF7,” “MAX,” “STAT1,” “STAT3,” “XBP1,” and “YY1,” which emerged from the modules that showed significant changes in expression patterns over the HIV progression stages (Ray and Bandyopadhyay, 2016).

Ding et al. (2019) created several co-expression networks based on transcription data from the CD4+ and CD8+ T cells as well as whole blood obtained from subjects with different viremic control statuses, e.g., viremic controllers and elite controllers. They found significant positive associations between several modules and clinical parameters describing HIV progression and the loss of viremic control. The genes from these modules participate in immune-related pathways and cellular processes such as type I interferon signaling pathway, complement activation, the positive regulation of B cell activation, cellular responses to stress, leukocyte migration, and responses to cytokines (Ding et al., 2019).

Zak et al. (2012) used the time-serial measurement of gene expression profiles in peripheral blood mononuclear cells, which were derived from subjects who were provided with MRKAd5/HIV vaccination. These researchers used modular analysis framework to deconvolute complex transcriptional profiles into functionally interpretable patterns. They performed pathway enrichment analysis of genes from revealed modules and found increased expression of genes associated with inflammation, interferon response, and myeloid cell trafficking, and decreases in lymphocyte-specific transcripts, which leads to the hypothesis that the vaccine was stimulating an influx of myeloid cells and an efflux of lymphoid cells from the circulation. The authors also compared the gene expression profiles between subjects who have different magnitudes of HIV-specific CD8+ T-cell responses to the vaccine, and they identified 209 DEGs that were associated with cytotoxic responses, including inhibitory killer cell Ig-like receptor KIR2DL1, the NK-cell activating receptor CLEC2D, and the NK-cell signaling adaptor EWS-FLI1-activated transcript 2 (EAT-2). This finding is important because the adenoviral expression of EAT-2 enhanced vaccine-induced T-cell responses as part of a vaccine strategy (Zak et al., 2012).

### Signed Network-Based Analysis

The signed network is a type of molecular network in which (1) all edges have a direction representing a signal flow from the source to the target node; and (2) all edges are either positive (standing for activation) or negative (representing inhibition). Signaling and gene regulatory networks are the most commonly used networks of this type (Csermely et al., 2013). The signaling network consists of signed direct interactions between proteins, RNAs, and secondary messengers, and it represents the signal flow from receptors to transcriptional factors or other effector molecules. The gene regulatory network consists of signed, mostly indirect interactions between genes, where edges represent how one gene can change the transcription of another gene, either up- or down-regulation (Csermely et al., 2013). The signaling pathways are usually manually created by experts based on a great deal of information regarding the PPIs, post-translational modifications, siRNA-based genetic knockdowns, and data types. Information about signaling pathways can be obtained from various databases such as KEGG^24^, Reactome^25^, NetPath^26^, SPIKE^27^, and Signor 2.0^28^. A gene regulatory network can be created using reverse engineering methods that are applied to gene transcription data perturbed multiple times, e.g., by siRNA to different genes or small molecule inhibitors (Csermely et al., 2013).

Signed networks can be used in HIV-related research for (1) identifying the motifs of directed interactions between HIV and human proteins (van Dijk et al., 2010; Biswas et al., 2019); and (2) creating dynamic models of HIV interaction with human cells (Oyeyemi et al., 2015; Bensussen et al., 2018).

Motifs are chains or contours of 3–6 vertices in a directed network that are much more common than they are in a random network. These building blocks have been used to study the structure and dynamic behavior of networks. van Dijk et al. (2010) identified several motifs consisting of human and HIV proteins, e.g., positive feedback, positive and negative co-regulation, co-activation motifs, activation, and inhibition cliques. These motifs may have an essential role in HIV-human interactions. For example, the three-node feedback loop motif, which was identified as indirect self-regulation, is a pattern in which an HIV protein regulates or signals a human protein that regulates/signals another HIV protein in turn (van Dijk et al., 2010).

The dynamic of HIV–host interactions can be simulated by creating discrete and continuous models. Oyeyemi et al. (2015) applied Boolean discrete modeling to the T-cell activation signaling pathway containing both HIV and human proteins. They found that the model reproduced the expected patterns of T-cell activation, co-stimulation, and co-inhibition. Through *in silico* knockouts, the model identified an additional nine HDFs, including members of the PI3K signaling pathway that are essential for viral replication. The revealed potential HDFs were retrospectively confirmed by comparison with the results of three functional genomic screens (Oyeyemi et al., 2015). Bensussen et al. (2018) created a gene regulatory network of latent proviruses in resting CD4+ T cells containing human and HIV proteins as well as HIV non-coding RNAs. They applied both Boolean and continuous mathematical models, which were based on ordinary differential equations, to simulate the latency reversion. The authors found that viral non-coding RNAs can counteract the activity of latency reversing agents, which may explain the failure of these compounds to reactivate the latent reservoirs of HIV. They also found that inhibitors of histone methyltransferases, together with releasers of the positive transcription elongation factor (P-TEFb), may increase proviral reactivation despite the self-repressive effects of viral non-coding RNAs (Bensussen et al., 2018).

## Conclusion

HIV/AIDS remains one of the most significant dangers for humankind. Although the existing antiretroviral therapy allows for the control of the virus and prevents transmission, HIV infection remains a global health problem due to the impossibility of eliminating the virus from the human body and problems with creation of anti-HIV vaccine. To overcome the limitations of the existing anti-HIV therapy, to improve its efficacy and safety, and to develop new therapeutic approaches, such as therapeutic and preventive vaccines as well as approaches to cure latent infection, a deeper understanding of the mechanisms of HIV-human interaction is required. A network-based analysis of OMICs data may shed light on the corresponding mechanisms and allows for the identification of new points in therapeutic interventions. To date, more than 2900 interactions between human and HIV-1 proteins belonging to different viral groups and subtypes have been added to public databases. Most of these interactions belong to HIV-1 group M subtype B, whereas other groups and subtypes are associated with very few or no interactions, which results in difficulties in the network-based analysis of other HIV variants. We found 232 HIV-related transcriptomics experiments in which the expression profiles of human and HIV coding and non-coding RNAs were measured in various cell types, *in vivo* and *in vitro*, under different conditions. Different individuals may have different levels of susceptibility to HIV, disease progression rates, and different responses to drug and vaccine treatments. The identification of DEGs between various conditions with the pathway enrichment analysis allows researchers to explain the differences in these conditions. Particularly, the comparison of transcriptional profiles in various immune cells from individuals treated with more or less effective vaccines, with stronger or weaker immune response to particular vaccine allows identifying genes and pathways, which modulation by adjuvants may increase vaccine efficacy. Similarly, comparison of transcriptional profiles between uninfected and latently infected cells allows identifying unknown mechanisms of latency, which may help to develop new more effective latency reversing agents or agents causing transcriptional silencing of integrated HIV genome (“shock and kill,” “block and lock” strategies). Comparison of transcriptional profiles between chronically infected individuals, viremic and elite controllers allows identifying mechanisms of decreased susceptibility to HIV infection. Mimicking the corresponding transcriptional profiles of viremic and elite controllers by various chemical and biological agents may increase the efficacy of antiretroviral therapy and vaccines. The creation of co-expression networks with the subsequent identification of dense gene clusters allows for the identification of new HIV-related pathway and cellular processes that cannot be obtained through a simple analysis of DEGs. In addition to protein-protein interactions and transcriptomics data, a few datasets on the interactions between human and viral RNAs, genomics, proteomics, epigenomics data as well as data from functional genomic screenings are currently available. The integration of human and human-HIV protein-protein interactions with other types of OMICs data allows for the creation of context-specific networks reflecting particular experimental and clinical conditions. Networks reflecting different degree of susceptibility to HIV infection (chronically infected patients, viremic and elite controllers), productive or latent HIV infection can be created. The identification of modules in human context-specific protein–protein interaction networks, as in co-expression network, allows for the identification of more HIV-related pathways and cellular processes than through simple comparison of transcriptomic and proteomic profiles. The analysis of the topology of context-specific human or human–HIV protein–protein interaction networks may assists in identifying the proteins with high degree or centrality, which are essential for HIV–human interaction, and may represent the most perspective human targets to prevent infection of human CD4+ cells, reactivate or silent latent HIV infection, and model the efficacy of immune response induced by vaccines.

Since there are hundreds of publicly available transcriptomic experiments that were performed under many conditions as well as thousands of known human and HIV-human protein-protein interactions, whereas only a small portion of them have been used in network-based analyses, this gap provides an opportunity to create many novel network-based models and potentially obtain new knowledge on HIV-human interaction mechanisms.

## Author Contributions

All authors listed have made a substantial, direct and intellectual contribution to the work, and approved it for publication.

## Conflict of Interest

The authors declare that the research was conducted in the absence of any commercial or financial relationships that could be construed as a potential conflict of interest.

## Footnotes

1 https://thebiogrid.org

2 https://www.ebi.ac.uk/intact

3 https://mint.bio.uniroma2.it

4 https://dip.doe-mbi.ucla.edu/dip/Main.cgi

5 https://string-db.org

6 https://www.ncbi.nlm.nih.gov/genome/viruses/retroviruses/hiv-1/interactions

7 https://www.uniprot.org

8 http://www.phisto.org

9 http://virhostnet.prabi.fr

10 https://virusmentha.uniroma2.it/about.php

11 http://viruses.string-db.org

12 http://www.rna-society.org/virbase/index.html

13 http://210.46.85.180:8080/vmireg

14 http://crdd.osdd.net/servers/virmirna

15 https://www.ebi.ac.uk/gwas

16 https://www.gwascentral.org

17 https://www.ncbi.nlm.nih.gov/gap

18 https://www.ncbi.nlm.nih.gov/geo

19 https://www.ebi.ac.uk/arrayexpress

20 http://geneontology.org

21 http://hivlatency.erc.monash.edu

22 http://www.proteomexchange.org

23 https://www.ebi.ac.uk/pride/archive

24 https://www.genome.jp/kegg

25 https://reactome.org

26 http://www.netpath.org

27 https://www.cs.tau.ac.il/~spike

28 https://signor.uniroma2.it
